# Multifunctional targeting daunorubicin plus quinacrine liposomes, modified by wheat germ agglutinin and tamoxifen, for treating brain glioma and glioma stem cells

**DOI:** 10.18632/oncotarget.2267

**Published:** 2014-07-26

**Authors:** Xue-Tao Li, Rui-Jun Ju, Xiu-Ying Li, Fan Zeng, Ji-Feng Shi, Lei Liu, Cheng-Xiang Zhang, Meng-Ge Sun, Jin-Ning Lou, Wan-Liang Lu

**Affiliations:** ^1^ State Key Laboratory of Natural and Biomimetic Drugs, School of Pharmaceutical Sciences, Peking University, Beijing, China; ^2^ School of Pharmacy, Liaoning University of Traditional Chinese Medicine, Dalian, China; ^3^ Institute of Clinical Medical Sciences, Chia-Japan Friendship Hospital, The Ministry of Health, Beijing, China

**Keywords:** multifunctional targeting liposomes, wheat germ agglutinin, daunorubicin, tamoxifen, brain glioma stem cells

## Abstract

Most anticancer drugs are not able to cross the blood-brain barrier (BBB) effectively while surgery and radiation therapy cannot eradicate brain glioma cells and glioma stem cells (GSCs), hence resulting in poor prognosis with high recurrence rates. In the present study, a kind of multifunctional targeting daunorubicin plus quinacrine liposomes was developed for treating brain glioma and GSCs. Evaluations were performed on in-vitro BBB model, murine glioma cells, GSCs, and GSCs bearing mice. Results showed that the multifunctional targeting daunorubicin plus quinacrine liposomes exhibited evident capabilities in crossing the BBB, in killing glioma cells and GSCs and in diminishing brain glioma in mice. Action mechanism studies indicated that the enhanced efficacy of the multifunctional targeting drugs-loaded liposomes could be due to the following aspects: evading the rapid elimination from blood circulation; crossing the BBB effectively; improving drug uptake by glioma cells and GSCs; down-regulating the overexpressed ABC transporters; inducing apoptosis of GSCs via up-regulating apoptotic receptor/ligand (Fas/Fasl), activating apoptotic enzymes (caspases 8, 9 and 3), activating pro-apoptotic proteins (Bax and Bok), activating tumor suppressor protein (P53) and suppressing anti-apoptotic proteins (Bcl-2 and Mcl-1). In conclusion, the multifunctional targeting daunorubicin plus quinacrine liposomes could be used as a potential therapy for treating brain glioma and GSCs.

## INTRODUCTION

Malignant brain glioma is the most lethal and aggressive type of cancer in oncology with a median survival of only 14.6 months and a 5-year survival rate of less than 5% [1, 2]. Treatment for brain glioma depends on the location, the cell type and the grade of malignancy. Usually, treatment is a combined approach, using surgery, radiation therapy and chemotherapy. However, the prognosis for patients is generally poor due to its pathological features. Most anticancer drugs are not able to cross the blood–brain barrier (BBB) effectively [3]. Furthermore, surgery and radiation therapy cannot eradicate brain glioma cells and glioma stem cells (GSCs), thus leading to high recurrence rates. Therefore, there is an urgent need for developing a new chemotherapy strategy to overcome the limitations.

The BBB is a highly selective permeability barrier, and formed by capillary endothelial cells that are connected by tight junctions, which are composed of transmembrane proteins, occludin, claudins, and junctional adhesion molecules, etc. This “barrier” restricts the passage of solutes and anticancer agents. Nevertheless, physiological substances could cross the BBB by passive diffusion of small molecules, such as water and oxygen; by transporter mediated transport of polar molecule nutrients, such as amino acids and glucose; and by receptor mediated transport of large endogenic molecules, such as insulin and hormone [4]. These suggest that the drug carrier with a suitable unlocking key may be able to cross the BBB by opening the “door”, while the targeting strategy offers a likely unlocking key.

This poor prognosis of brain glioma is also due to therapeutic resistance and tumor recurrence after surgical removal. Moreover, a relatively quiescent subset of endogenous glioma cells, i.e., GSCs, is responsible for relapse through the production of transient populations of highly proliferative cells [5]. GSCs being dormant state are highly drug-resistant to regular chemotherapies and make cell cycle specific anticancer drugs in vain. Furthermore, GSCs highly express ATP-binding cassette transporters (ABC transporters), which efflux the internalized drugs from the GSCs.

Wheat germ agglutinin (WGA) is a lectin that protects wheat from insects, yeast and bacteria. An agglutinin protein binds to N-acetyl-D-glucosamine and sialic acid, and N-acetyl-D-glucosamine in the natural environment of wheat is found in the chitin of insects, and the cell membrane of yeast and bacteria [6]. Investigations reveal that WGA has the potential to transfer drug across the BBB mediated by adsorptive endocytosis [7]. In addition, WGA can specifically bind to membrane-associated glycoproteins highly expressed on cancer and cancer stem cells [8]. These suggest that WGA may be a suitable targeting ligand to transport drug carriers across the BBB and further to target glioma cells and GSCs.

Tamoxifen (TAM) is an antagonist of estrogen receptor in breast tissue but an agonist in other tissues such as the endometrium. It is used in the conventional anti-estrogen therapy for hormone receptor-positive breast cancer in pre-menopausal women [9]. TAM has shown the capability to circumvent multi-drug resistance (MDR) by blocking drug efflux due to overexpressed ABC transporters in cancer cells [10]. Our previous studies also demonstrated that TAM could be used for improving the transport of drug across the BBB [11].

Daunorubicin is widely used in treating a variety of cancers [12]. The underlying mechanism that daunorubicin takes effects is mainly by interfering DNA and RNA synthesis in the cellular nuclei [13]. Quinacrine has been used as an antiprotozoal, antirheumatic and an intrapleural sclerosing agent. Previous reports have demonstrated that quinacrine could potentially induce apoptosis in many tumor cells, including cervical carcinoma [14], gastric cancer [15], and neck squamous carcinoma [16]. Besides, quinacrine has also shown the capability to induce apoptosis of breast cancer stem cells [17].

Therefore, we hypothesized that the multifunctional targeting daunorubicin plus quinacrine liposomes modified with WGA and TAM could be able to cross the BBB, and kill glioma cells and GSCs. In the liposomes, TAM was incorporated into the lipid bilayer for blocking the drug efflux transporters which overexpressed on the BBB and GSCs, and WGA was modified on the surface of the liposomes for crossing the BBB and further targeting glioma cells and GSCs. Daunorubicin and quinacrine were entrapped into the liposomes as anticancer drug and an apoptosis-inducing agent, respectively. The objectives of the present study were to construct the multifunctional targeting liposomes, and to characterize the anticancer efficacy for treating brain glioma and GSCs.

## RESULTS

### Characterization of the multifunctional targeting liposomes

**Fig.1:**
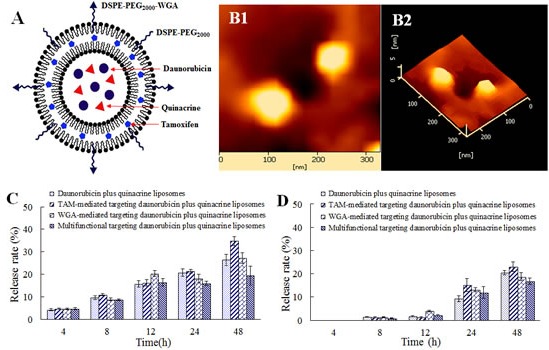
Characterization of multifunctional targeting daunorubicin plus quinacrine liposomes A. A schematic representation of the multifunctional targeting daunorubicin plus quinacrine liposomes; B. AFM images of the multifunctional targeting daunorubicin plus quinacrine liposomes (B1, two-dimension image; B2, three-dimension image); C. Release rates of daunorubicin; D. Release rates of quinacrine. Data are presented as mean ± standard deviation (SD, n=3).

Fig. 1A and 1B show schematic drawing and an atomic force microscopy (AFM) of multifunctional targeting drugs-loaded liposomes, respectively. Results demonstrated that the multifunctional targeting liposomes were round shaped with smooth surface. Fig. 1C and 1D show the release rates of daunorubicin and quinacrine from varying liposomal formulations. Results exhibited that the release rates of daunorubicin and quinacrine from these liposomes were less than 5% within the initial 4 h. For multifunctional targeting drugs-loaded liposomes, the *in vitro* release rate of daunorubicin was 19.46 ± 4.25% at 48 h, and that of quinacrine was 16.80 ± 1.37% at 48 h. The amount of WGA molecules presented in one liposome particle was calculated to be 1.26 × 10^3^.

Table 1 lists the characterization of the multifunctional targeting drugs-loaded liposomes. In all types of liposomes, the encapsulation efficiencies of daunorubicin or quinacine were >90%, respectively. The coupling rates of TAM and WGA were > 90% and > 88%, respectively. Average particle sizes of all liposomes were approximately in the range of 100-106 nm with a narrow polydispersity index each (≤ 0.22), and the charge values were slightly negative.

**Table 1:** Characterization of liposomes

	Encapsulation efficiency (%)		Modifying efficiency (%)		Particle size (nm)	PDI	Zeta potential (mV)
	Daunorubicin	Quinacrine	Tamoxifen	WGA			
Blank multifunctional targeting liposomes	-	-	95.32±2.42	90.34±1.13	100.77±3.62	0.219±0.014	−0.065±0.021
Daunorubicin plus quinacrine liposomes	96.04±1.07	96.34±3.00	-	-	103.97±2.05	0.177±0.016	−0.102±0.030
TAM-mediated targeting daunorubicin plus quinacrine liposomes	95.18±2.10	95.13±4.76	97.20±3.29	-	105.93±3.73	0.168±0.012	−0.038±0.041
WGA-mediated targeting daunorubicin plus quinacrine liposomes	91.47±0.76	97.30±3.12	-	88.73±0.90	104.43±2.70	0.183±0.006	−0.053±0.060
Multifunctional targeting daunorubicin plus quinacrine liposomes	94.05±2.57	94.84±4.02	93.58±3.66	88.67±1.48	104.05±3.56	0.175±0.007	−0.089±0.057

### Identification of glioma stem cells

**Fig.2:**
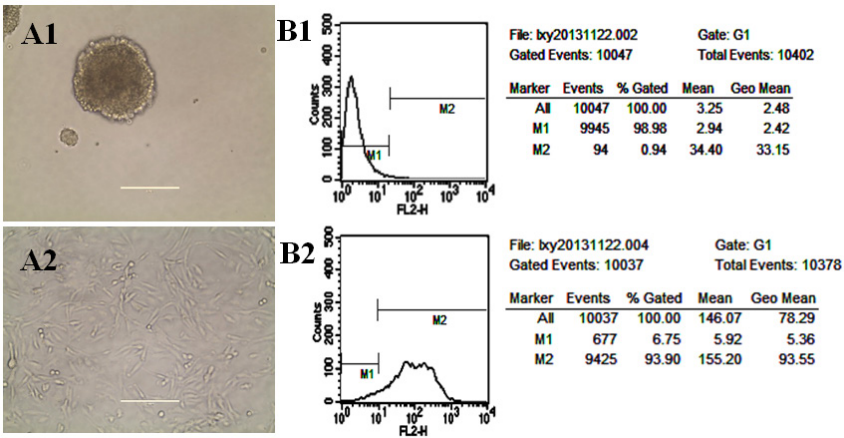
Identification of glioma stem cells (GSCs) A1. Image of GSCs spheroids cultured in serum-free medium; A2. Image of adherent GSCs cultured in serum-containing medium; B1. GSCs treated as isotype controls; B2. GSCs stained with anti-mouse/rat nestin-phycoerythrin antibiodies.

Fig. 2A1 and 2A2 show images of GSCs spheroids cultured in serum-free medium and adherent GSCs cultured in serum-containing medium, respectively. Fig. 2B1 and 2B2 indicate the phenotypes of GSCs. Results showed that GSCs spheroids cultured in serum-free medium for three weeks displayed high level of nestin (marked as nestin^+^). Purity of GSCs dissociated from the spheroids in serum-free culture medium was 93.90% by compared with isotype control.

### Uptake and distribution in glioma cells and glioma stem cells

**Fig.3:**
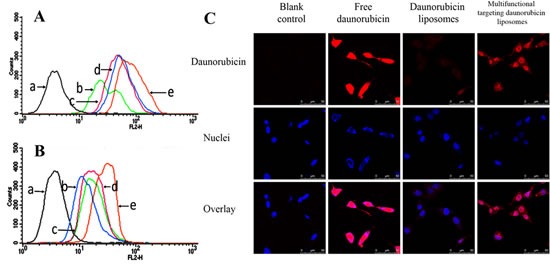
Cellular uptake and distribution in GSCs A. Cellular uptake of glioma cells. B. Cellular uptake of GSCs. C. Laser scanning confocal microscopy images of GSCs incubated with varying formulations. a. Blank control; b. Daunorubicin liposomes; c. TAM-mediated targeting daunorubicin liposomes; d. WGA-mediated targeting daunorubicin liposomes; e. Multifunctional targeting daunorubicin liposomes.

Fig. 3A and 3B show the cellular uptake by glioma cells and by GSCs after incubation with varying formulations. Results showed that the ranks of cellular uptake by glioma cells or by GSCs were multifunctional targeting daunorubicin liposomes > WGA-mediated targeting daunorubicin liposomes > TAM-mediated targeting daunorubicin liposomes > daunorubicin liposomes.

Fig. 3C illustrates confocal images for distribution of daunorubicin into GSCs after incubation with varying formulations. Results showed that the multifunctional targeting daunorubicin liposomes exhibited much higher fluorescence intensity in GSCs, indicating that much amount of drug had been internalized by the GSCs. Besides, the most intensive fluorescence was observed after treatment with free daunorubicin by direct interacting with the cells.

### Inhibiting glioma cells and glioma stem cells

**Fig.4:**
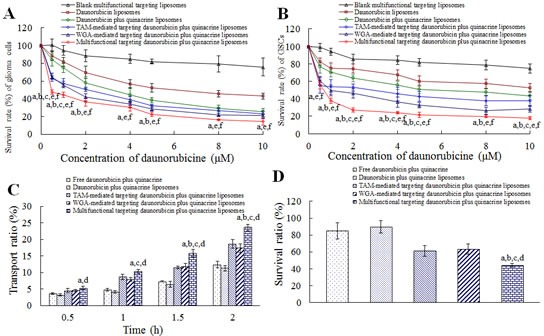
Eliminating brain glioma along with GSCs *in vitro* and multifunctional targeting effects in the BBB model A. Inhibitory effects to glioma cells; B. Inhibitory effects to GSCs; C. Transport ratio of daunorubicin across the BBB; D. Inhibitory effects to GSCs after crossing the BBB. Data are presented as mean ± SD (n=6). p < 0.05; a, vs. daunorubicin plus quinacrine liposomes; b, vs. TAM-mediated targeting daunorubicin plus quinacrine liposomes; c, vs. WGA-mediated targeting daunorubicin plus quinacrine liposomes; d, vs. free daunorubicin plus quinacrine; e, vs. daunorubicin liposomes; f, vs. blank multifunctional targeting liposomes.

Fig. 4A and 4B illustrate the inhibitory effects to glioma cells and GSCs after treatments with varying formulations. Among the six types of liposomes, multifunctional targeting drugs-loaded liposomes exhibited the strongest inhibitory effects to both glioma cells and GSCs at varying dose levels. In addition, the blank multifunctional targeting liposomes showed slight inhibitory effects to glioma cells and GSCs. The rank of IC50 values on either glioma cells or GSCs was daunorubicin liposomes > daunorubicin plus quinacrine liposomes > TAM-mediated targeting daunorubicin plus quinacrine liposomes > WGA-mediated targeting daunorubicin plus quinacrine liposomes > multifunctional targeting daunorubicin plus quinacrine liposomes (supplementary Table S1).

### Multifunctional targeting effects

Fig. 4C and 4D represent the multifunctional targeting effects of transporting across the BBB and targeting GSCs after treatments with varying formulations. The transendothelial electrical resistance (TEER) values of the BBB model were 285-299 Ω·cm^2^. Results showed that the multifunctional targeting drugs-loaded liposomes had the strongest transporting effects across the BBB at varying time-points in a time-dependent way. To further understand the multifunctional targeting effects across the BBB, the inhibitory effects to GSCs after crossing the BBB was measured. As shown in Fig. 4D, the rank of inhibitory effects to GSCs was multifunctional targeting drugs-loaded liposomes > TAM-mediated targeting drugs-loaded liposomes > WGA-mediated targeting drugs-loaded liposomes > free drugs > drugs-loaded liposomes.

### Regulating effects on ABC transporters and apoptotic proteins

**Fig.5:**
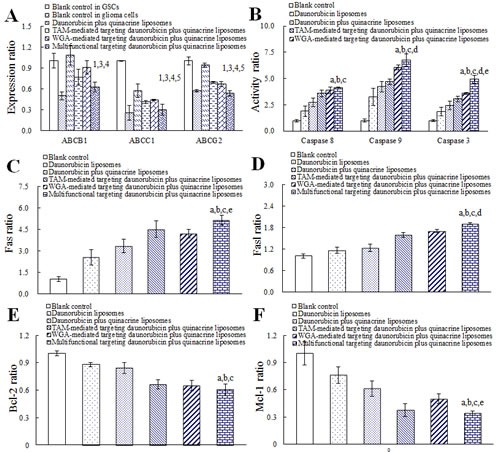

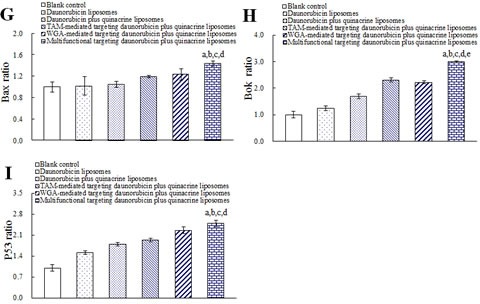
Regulating effects on the ATP-binding cassette transporters (ABCB1, ABCC1 and ABCG2), apoptotic enzymes (caspase 8, 9 and 3), apoptotic receptor/ligand (Fas and Fasl), anti-apoptotic proteins (Bcl-2 and Mcl-1), pro-apoptotic proteins (Bax and Bok), and P53 protein in GSCs after treatments with varying formulations A. ABC transporters; B. Caspase enzymes; C. Fas; D. Fasl; E. Bcl-2; F. Mcl-1; G. Bax; H. Bok; I. P53. Data are presented as the mean ± SD (n=3). p < 0.05; 1, vs. blank control in GSCs; 2, vs. blank control in glioma cells; 3, vs. daunorubicin plus quinacrine liposomes; 4, vs. TAM-mediated targeting daunorubicin plus quinacrine liposomes; 5, vs. WGA-mediated targeting daunorubicin plus quinacrine liposomes; a, vs. blank control; b, vs. daunorubicin liposomes; c, vs. daunorubicin plus quinacrine liposomes; d, vs. TAM-mediated targeting daunorubicin plus quinacrine liposomes; e, vs. WGA-mediated targeting daunorubicin plus quinacrine liposomes.

Fig. 5A exhibits expression levels of ABC transporters in glioma cells and GSCs before and after treatments with varying formulations. Before drug treatments, the expression ratios of drug-resistant ABC transporters in glioma cells (ABCB1 = 0.50 ± 0.06, ABCC1 = 0.26 ± 0.10, and ABCG2 = 0.57 ± 0.01) were evidently lower than those in GSCs (ABCB1, ABCC1 or ABCG2 = 1, respectively). After treatment with multifunctional targeting drugs-loaded liposomes for 24 h, expression ratios of ABCB1, ABCC1 and ABCG2 in GSCs were lowered to be 0.63 ± 0.07, 0.30 ± 0.08 and 0.53 ± 0.04, respectively.

Fig. 5B illustrates the activated apoptotic enzymes caspase 8, 9 and 3 in GSCs after treatments with varying formulations. Compared with blank control, activity ratios of caspase 8, 9 and 3 were enhanced to 4.11 ± 0.08, 6.75 ± 0.59 and 4.93 ± 0.39 folds after treatment with multifunctional targeting drugs-loaded liposomes, respectively. Among all groups, the multifunctional targeting drugs-loaded liposomes exhibited the most significant activating effects on caspase 8, 9 and 3.

Fig. 5C, D, E, F, G, H, and I display the regulating effects on the apoptotic proteins in GSCs after treatments with varying formulations. Compared with the control formulations, multifunctional targeting drugs-loaded liposomes significantly increased the activity ratios of apoptotic receptor (Fas in Fig. 5C) and ligand (Fasl in Fig. 5D), down-regulated the activity ratios of anti-apoptotic proteins (Bcl-2 in Fig. 5E and Mcl-1 in Fig. 5F); and up-regulated the activity ratios of pro-apoptotic proteins (Bax in Fig. 5G and Bok in Fig. 5H) and tumor suppressor protein (P53 in Fig. 5I).

### Penetrating ability and destructing effects in glioma stem cell spheroids

**Fig.6:**
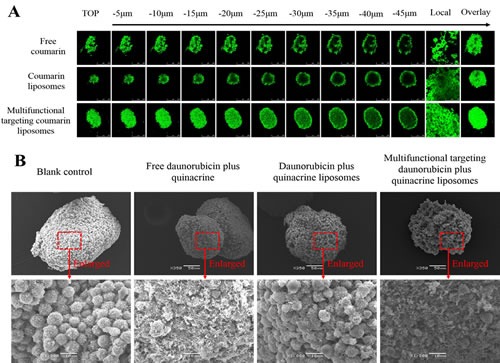
Penetrating ability and destructing effects in GSCs spheroids A. Confocal images at different layers from top to middle of the GSCs spheroids; B. SEM photographs of GSCs spheroids.

Fig. 6A shows the penetrating ability into GSCs spheroids after treatments with varying formulations. Results showed that the rank of fluorescent intensities in the spheroids were multifunctional targeting coumarin liposomes > free coumarin ≥ coumarin liposomes. Fig. 6B represents the destructing effects to GSCs spheroids after treatments with varying formulations. The rank of destructing effects to the spheroids were multifunctional targeting daunorubicin plus quinacrine liposomes > free daunorubicin plus quinacrine > daunorubicin plus quinacrine liposomes.

### Anticancer efficacy and *in vivo* imaging

**Fig.7:**
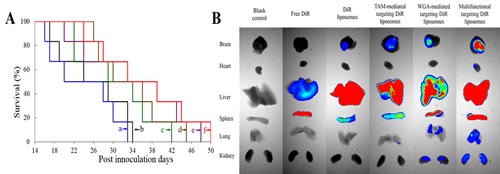

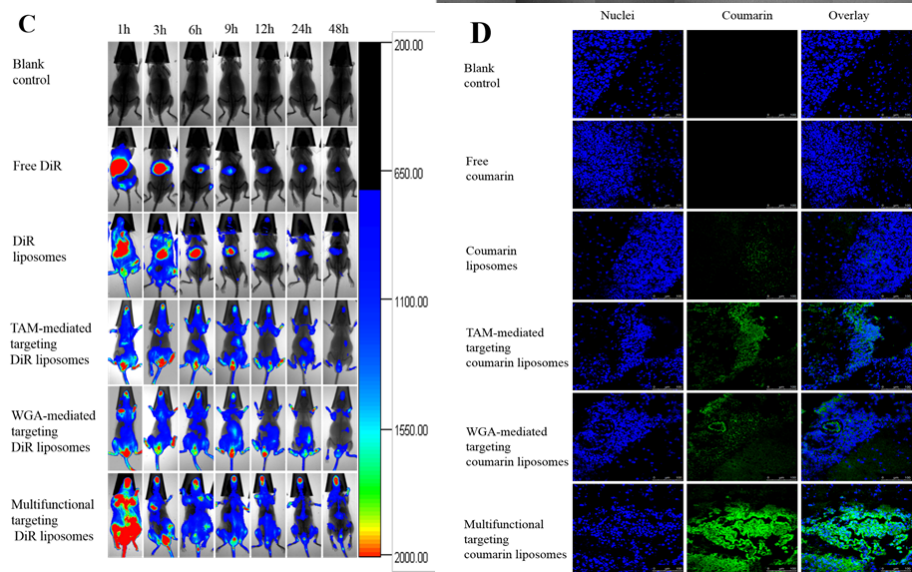
Anticancer efficacy, in vivo real-time imaging observation and the specificity to glioma tissues in the intracranial glioma-bearing ICR mice after treatment with varying formulations A. Kaplan-Meier survival curves; B. Ex vivo optical images of tumor masses and organs at 48 h; C. *In vivo* real-time images. D. The specificity to glioma tissues. a, saline; b, free daunorubicin plus quinacrine; c, daunorubicin plus quinacrine liposomes; d, TAM-mediated targeting daunorubicin plus quinacrine liposomes; e, WGA-mediated targeting daunorubicin plus quinacrine liposomes; f, multifunctional targeting daunorubicin plus quinacrine liposomes.

Fig. 7A represents Kaplan-Meier survival curves of brain glioma-bearing mice arising from GSCs. The median survival time were 26.00, 24.00, 30.83, 34.50, 33.67, and 36.33 days after treatments with saline, free daunorubicin plus quinacrine, daunorubicin plus quinacrine liposomes, TAM-mediated targeting daunorubicin plus quinacrine liposomes, WGA-mediated targeting daunorubicin plus quinacrine liposomes and multifunctional targeting daunorubicin plus quinacrine liposomes, respectively (supplementary Table S2). The survival ranges of mice treated with multifunctional targeting daunorubicin plus quinacrine liposomes (24-50 days) were significantly longer than those treated with saline (17-34 days).

Fig. 7B illustrates the ex vivo optical images of tumor masses and major organs after the tumor-bearing mice were sacrificed at 48 h. After injection of free 1, 1-dioctadecyl-3,3,3,3-tetramethylindotricarbocyanine iodide (DiR) at 48 h, the fluorescent signals could not be detected in heart, lung, kidney and brain, but was observed in liver and spleen. After injection of DiR liposomes at 48 h, the fluorescent signals were weakly observed in brain. After injection of multifunctional targeting DiR liposomes at 48 h, the fluorescent signals were observed in brain, liver and spleen, showing the strongest fluorescent signals.

Fig. 7C depicts the real-time distribution and accumulation of multifunctional targeting DiR liposomes in brain glioma-bearing mice. After injection of multifunctional targeting DiR liposomes, a strong DiR fluorescent signal was observed in the whole blood circulatory system at the early stage, and maintained up to 48 h in the tumor tissue. By contrast, after administration of free DiR, the fluorescent signal was rapidly distributed in the liver at 3 h, and gradually weakened or disappeared after 9 h. The rank of DiR fluorescent signal at the designated time-point was multifunctional targeting DiR liposomes > WGA-mediated targeting DiR liposomes ≥ TAM-mediated targeting DiR liposomes > DiR liposomes > free DiR.

Fig. 7D depicts the specificity to brain glioma tissues after treatments with varying formulations. Results showed that a strong green fluorescent signal was observed in tumor tissues after treatment with multifunctional targeting coumarin liposomes. By contrast, the fluorescent signal was too weak to be distinguished in tumor tissues after administration of free coumarin. The rank of green fluorescent signal in tumor tissues was multifunctional targeting coumarin liposomes > WGA-mediated targeting coumarin liposomes ≥ TAM-mediated targeting coumarin liposomes > coumarin liposomes > free coumarin.

## DISCUSSION

Malignant brain glioma, being usually recrudescent after resection, is the most lethal and aggressive type of cancer in oncology [18, 19]. Results of previous studies show that chemotherapy does not significantly prolong the survival of patients with brain glioma because of the existence of BBB, which restricts the movement of most molecules and anticancer drugs from blood to brain [20, 21]. Another reason is the existence of the GSCs, which cannot be eradicated completely by surgery and radiation therapy. Consequently, a kind of multifunctional targeting daunorubicin plus quinacrine liposomes was developed in the present study aimed at crossing the BBB, and eliminating glioma cells and GSCs.

In the multifunctional targeting daunorubicin plus quinacrine liposomes, WGA is coupled on the surface of the liposomes by conjugating with DSPE-PEG_2000_-NHS, and TAM is inserted into the phospholipid bilayer as the multifunctional targeting ligands. Daunorubicin and quinacrine are encapsulated in the aqueous core of the liposome. Physicochemical characterizations of the multifunctional targeting liposomes are performed on the following aspects: high encapsulation efficiencies, small and well-distributed particle size (Table 1), smooth surface (Fig. 1B1 and 1B2), and delayed drug release (Fig. 1C and 1D). The high encapsulation efficiencies of the multifunctional targeting liposomes permit more drugs to be transported across the BBB with the functionalized carriers. The particle size of the liposomes can be beneficial for passive delivery to tumor interstitium through the leaky vasculature with 100-600 nm pores in the aggressive tumors by an enhanced permeability and retention (EPR) effect [22]. The delayed drug release will be beneficial for preventing rapid leakage during the process of drug delivery and increasing accumulation of the drug into the tumor masses.

In this study, GSCs are cultured and exist as spheroids in serum-free medium, and the spheroids are proven to have stem-like characteristics with high level of nestin (marked as nestin^+^, 93.55%) as compared with isotype control (Fig. 2B1 and 2B2). The targeting effects of multifunctional targeting liposomes are evidenced in the cellular uptake by flow cytometry (Fig. 3A and 3B), and the cellular distribution by confocal observation (Fig. 3C). The semi-quantitative flow cytometry demonstrates that the addition of WGA and TAM can significantly promote cellular uptake of the liposomes in both glioma cells and GSCs. The cellular distribution of multifunctional targeting liposomes is observed with a confocal method. Results show that the multifunctional targeting daunorubicin liposomes exhibit stronger purple fluorescence compared with daunorubicin liposomes. This phenomenon can be associated with the receptor-mediated endocytosis by WGA, which is modified onto the liposomal surface and displays high binding specificity with the sugar moiety of GSCs. The confocal results of targeting effects are consistent with those observed by cellular uptake.

In cytotoxic assays, multifunctional targeting daunorubicin plus quinacrine liposomes exhibit the strongest inhibitory effects on both glioma cells and GSCs (Fig. 4A and 4B). This is attributed to an enhanced cellular uptake of the targeting liposomes. Compared with daunorubicin liposomes, stronger inhibitory effects are also observed on glioma cells and GSCs after treatment with daunorubicin plus quinacrine liposomes, indicating that quinacrine can enhance anticancer effects possibly by inducing apoptosis of cancer cells.

In the study, BMVECs are used to build the BBB model, and the tight junctions are evaluated by TEER values [23]. TEER values are monitored during the whole process of experiments to make sure there is no leakage, thus guaranteeing accuracy of the BBB model. Results indicate that the liposomes modified with TAM, WGA or both evidently improve transport of drugs across the BBB (Fig. 4C and 4D).

A family of ABC transporters have been characterized to act specific physiological functions such as ABCA1 for cholesterol efflux onto high-density lipoproteins, while only a few of ABC transporters are identified to be responsible for multidrug resistance by efflux of drug from cancer cells, such as ABCB1, ABCC1 and ABCG2, etc. [24]. In the present study, these drug efflux-pump transporters are inferred to be associated with drug-resistance of GSCs, and accordingly, are selected for experimental observation. The results display that the expression ratios of the ABC transporters are distinctly different between glioma cells and GSCs before drug treatments, and the expression ratios of ABCB1, ABCC1 and ABCG2 in GSCs are down-regulated significantly after treatment with multifunctional targeting drugs-loaded liposomes (Fig. 5A).

Analyses of apoptotic enzymes demonstrate that after treatment with multifunctional targeting drugs-loaded liposomes, a cascade of apoptotic reactions happens via caspase-related signaling pathways (Fig. 5B). Apoptotic proteins (Fas, Fasl, Bax, Bok, Bcl-2, Mcl-1, and P53) in GSCs after treatments with varying formulations are measured, and results further elucidate that the apoptotic receptor/ligand (Fas/Fasl), pro-apoptotic proteins (Bax, Bok), anti-apoptotic proteins (Bcl-2, Mcl-1) and tumor suppressor protein (P53) are involved in the apoptotic process of GSCs. Consequently, this study suggests that there are the two ways in which GSCs die: killing by cytotoxic agents or inducing to commit suicide, i.e., apoptosis, by multifunctional targeting drug-loaded liposomes [25]. Since GSCs are resistant to the substrate of cytotoxic agents such as daunorubicin, inducing apoptosis can be an effective approach to eradicate the quiescent subsets in cancer populations.

Accordingly, the enhanced inhibitory effects to GSCs after treatment with multifunctional targeting drugs-loaded liposomes could be explained by the following facts: the activated Fas receptor (Fas) forms the death-inducing signaling complex (DISC) by binding with the ligand (Fasl), and the DISC is internalized via the cellular endosomal machinery [26]. This allows Fas-associated death domain (FADD) to bind the death domain (DD), and then caspase 8 (FADD-like) self-activate through proteolytic cleavage. The activated up-stream initiator caspase 8 catalyzes the cleavage of pro-apoptotic proteins (Bok and Bax) or inhibits activities of anti-apoptotic proteins (Bcl-2 and Mcl-1), hence resulting in translocation of the outer mitochondrial membrane and further cleavage of caspase 9. In case of activating the apoptotic enzymes (caspase 8 and 9), they will result in a cascade of apoptotic reactions, followed by activating the down-stream effector (caspase 3). Meanwhile, the activated P53 up-regulates the apoptotic receptor/ligand (Fas/Fasl), pro-apoptotic proteins (Bax, Bok) and apoptotic enzymes (caspases 8, 9 and 3) but down-regulates anti-apoptotic proteins (Bcl-2, Mcl-1). This eventually leads to DNA degradation, membrane blebbing, and other hallmarks of apoptosis [27].

The multifunctional targeting drugs-loaded liposomes also show a strong penetrating ability into GSCs spheroids and a destructing effect to the spheroids. By viewing confocal images at different layers of the spheroids from top to middle, the strongest fluorescent intensity can be observed after treatment with multifunctional targeting coumarin liposomes (Fig. 6A). Fluorescence overlay of different layers in GSCs spheroids also indicates that the multifunctional targeting liposomes have a strong penetrating ability. Furthermore, multifunctional targeting daunorubicin plus quinacrine liposomes display the most significant destructing effects to tumor spheroids compared with other formulations (Fig. 6B). The tightly organized spheroids after treatment with multifunctional targeting daunorubicin plus quinacrine liposomes become disintegrated and shrunken. Besides, a strong destructing effect happens as well after treatment with free daunorubicin plus quinacrine due to the direct exposure of free drugs.

As for evaluating the *in vivo* anticancer efficacy, brain glioma-bearing mice are made by inoculating GSCs. The survival effects demonstrate that the multifunctional targeting drugs-loaded liposomes have a strong potential in treating the brain glioma-bearing mice (Fig. 7A). The evident anticancer efficacy in treating brain glioma in mice is explained by the following aspects: (i) PEG chain of DSP-PEG_2000_ can avoid the rapid uptake by reticuloendothelial system (RES) and increase accumulation in tumor sites by EPR effect; (ii) WGA conjugated on the multifunctional targeting liposomal surface can enhance the transport of drugs-loaded liposomes across the BBB and the cellular uptake by GSCs; (iii) TAM inserted into the liposomal bilayer can enhance the transport of drugs across the BBB and circumvent drug efflux of ABC transporters overexpressed on the BBB and cellular membrane of GSCs; (iv) incorporated quinacrine can induce apoptosis of glioma cells and GSCs.

For understanding the distribution status of multifunctional targeting daunorubicin plus quinacrine liposomes *in vivo*, real-time imaging is performed in brain glioma-bearing mice. The near-infrared fluorescent probe DiR is encapsulated into the targeting liposomes for indicating the distribution. The imaging results *in vivo* demonstrate that the multifunctional targeting DiR liposomes have a long-lasting stability in blood circulation, and exhibit a significant accumulation in tumor masses (Fig. 7C). The ex vivo fluorescent images of tumor masses and major organs further confirm above distributions of the multifunctional targeting liposomes (Fig. 7B).

The specificity of the multifunctional targeting liposomes to glioma tissues is evidenced in tumor-bearing mice with a confocal method (Fig. 7D). Results show that the multifunctional targeting coumarin liposomes exhibit the strongest green fluorescence among varying formulations. This phenomenon is associated with the enhanced transport drugs across the BBB and the increased cellular uptake in both glioma cells and GSCs by WGA and TAM. The confocal results of specificity to tumor masses are consistent with those observed by *in vivo* real-time image.

## MATERIALS AND METHODS

### Preparation of the liposomes

Blank multifunctional targeting liposomes were prepared using the film dispersion method [28]. Briefly, egg phosphatidylcholine, cholesterol, polyethylene glycol-distearoylphosphatidylethanolamine (DSPE-PEG_2000_, NOF Corporation, Japan), N-hydroxysuccinimidyl-PEG_2000_-DSPE (DSPE-PEG_2000_-NHS, NOF Corporation, Japan) and TAM (Nanjing Tianzun Zezhong Chemicals, Co., Ltd, Nanjing, China) (60: 40: 2.5: 1.25: 8, molar ratio) were dissolved in chloroform in a pear-shaped flask. The chloroform was evaporated by a rotary evaporator, and the formed lipid film was hydrated with 250 mM ammonium sulfate by sonication in a water bath for 5 min. Subsequently, the suspensions were treated by a probe-type sonicator for 10 min (200 W). The suspensions were successively extruded through polycarbonate membranes (Millipore, Bedford, MA, USA) with the pore size of 400 nm and 200 nm for 3 times, respectively. Then, blank liposomes were obtained. The blank liposomes were further dialyzed (cut-off MW, 12,000-14,000) in the Hepes buffered saline (25 mM Hepes/ 150 mM NaCl) for 24 h. After dialysis, a certain amount of WGA (Shanghai BioSun Sci & Tech Co., Ltd, Shanghai, China) (DSPE-PEG_2000_-NHS: WGA = 30: 1, molar ratio) was added, followed by 10 h incubation at room temperature, and then a chromatographic separation (Sephadex G-100, Beijing Biodee Biotechnology Co., Ltd. Beijing, China) was performed to remove the unbound WGA. A volume of 5 mL liposome suspensions was mixed with the solution of daunorubicin (Nanjing Tianzun Zezhong Chemicals, Co., Ltd, Nanjing, China) and quinacrine (Sigma- Aldrich Corporation Beijing local agent, China) (daunorubicin: quinacrine = 1: 1, molar ratio; lipids: drug = 15: 1, w/w). After mixing, the suspensions were incubated at 40 °C in a water bath, and intermittently shaken for 20 min, thus producing the multifunctional targeting daunorubicin plus quinacrine liposomes [29].

WGA-mediated targeting daunorubicin plus quinacrine liposomes, TAM-mediated targeting daunorubicin plus quinacrine liposomes and daunorubicin plus quinacrine liposomes were prepared using the same procedures as above, excluding the addition of TAM, WGA or both. The multifunctional targeting coumarin liposomes or coumarin liposomes (lipids: coumarin = 200: 1, w/w) and the multifunctional targeting DiR liposomes or DiR liposomes (lipids: DiR = 300: 1, w/w) were similarly prepared as the fluorescent probes [30].

### Characterization of the liposomes

Particle size, polydispersity index (PDI) and zeta potential values were measured using a Nano Series Zen 4003 Zetasizer (Malvern Instruments Ltd, Malvern, UK). Morphology of the liposomes was observed by AFM (SPI3800N series SPA-400, NSK Ltd., Tokyo, Japan). The drugs-loaded liposomes were separated from un-encapsulated drugs by passing the liposomes over a Sephadex G-50 column eluted with 20 folds HBS buffer (v/v). Daunorubicin, quinacrine and TAM were measured by HPLC (Agilent Technologies Inc., Cotati, CA, USA), respectively. Encapsulation efficiencies (EE) of daunorubicin, quinacrine and TAM were calculated using the formula: EE = (W_encap_ / W_total_) ×100%, where W_total_ and W_encap_ were the measured amounts of drugs in the liposome suspensions before and after passing over the Sephadex G-50 column, respectively [31]. WGA was measured by the BCA protein assay kit (Pierce Corporation, Beijing local agent, China). WGA coupling rate was calculated by the ratio of the WGA amount on the liposomes after separation to the added amount.

*In vitro* release of daunorubicin and quinacrine in drugs-loaded liposomes was performed by the dialysis against the release medium (PBS containing 10% fetal calf serum). Daunorubicin and quinacrine in the samples were measured by HPLC as above, and release rates of both drugs were estimated as our previous reports [32, 33]. Each assay was repeated in triplicate.

In order to quantify the amount of WGA molecules present in one liposome particle, the density of one liposome particle was assumed to be 1.2 g/cm^3^ according to the reference [34] and the number of WGA per liposome was calculated with the following equation:

N_WGA_= (N_total_ × MOL_WGA_%×NA)/(M_total_ /M)

Where N_WGA_ represents the amount of WGA in one liposome; N_total_ is the total mole number of lipid materials in a sample; MOL_WGA_% is the mol% of WGA molecules in the total lipid materials used; NA is Avogadro's constant; M_total_ is the total mass of liposomes in the same sample; M is the average mass of one liposome calculated with the measured average particle diameter and density (1.2g/cm^3^).

### Cell culture and identification of glioma stem cells

Glioma cells (Institute of Basic Medical Science, Chinese Academy of Medical Science, Beijing, China) were maintained in Ham's F10 medium (Macgene) supplemented with 10% fetal bovine serum (FBS, Gibco). GSCs were grown in serum-free DMEM-F12 (Macgene) supplemented with 20 ng/mL basic FGF, 20 ng/mL EGF (Macgene) and 2% B27 (Gibco). GSCs were identified with the following procedures [35]. Brieﬂy, GSCs spheroids being cultured in serum-free medium under 5% CO_2_ at 37 °C for three weeks were collected, enzymatically dissociated and washed in PBS. The samples were fixed with 4% paraformaldehyde for 10 min. After being penetrated with 0.2% Triton-X100, the cells were incubated with monoclonal anti-mouse/rat nestin-phycoerythrin or their appropriate isotype controls (R&D Systems Inc, USA) for 30 min in the dark. The samples were then washed 3 times and re-suspended in 500 μL cold PBS. Then, they were performed on a FACScan ﬂow cytometer (Becton Dickinson, USA) [36].

### Uptake and distribution in glioma and glioma stem cells

Cellular uptakes of multifunctional targeting drugs-loaded liposomes by glioma cells and by GSCs were semi-quantified. Glioma cells and single cell suspensions of GSCs were seeded into 6-well culture plates at a density of 4 × 10^5^ cells/well and incubated for 24 h, and then the cells were treated with varying drug formulations at a concentration of 10 μM daunorubicin. Culture medium was used as a blank control. After incubation for 3 h, the cells were washed 3 times with PBS, trypsinized and harvested in 300 μL PBS. Fluorescence intensity of daunorubicin was analyzed by flow cytometry with 1 × 10^4^ cells collected. The excitation wavelength of daunorubicin was set at 488 nm and the emission wavelength was in the range 560 - 590 nm. Each assay was repeated in triplicate.

To observe cellular distribution of multifunctional targeting drugs-loaded liposomes, GSCs were seeded into chambered coverslips at a density of 2×10^5^ cells/dish and incubated for 24 h. Then, varying drug formulations were applied to the dishes at a concentration of 10 μM daunorubicin and incubated for another 3 h. Control group was performed with blank medium. The cells were then washed 3 times with PBS, fixed with 4% paraformaldehyde for 10 min and stained with Hochest 33258 (2 μg/mL) for 5 min. The samples were analyzed using a confocal laser scanning fluorescent microscopy (Leica, Heidelberg, Germany) [37].

### Inhibiting glioma cells and glioma stem cells

Glioma cells and GSCs were seeded into 96-well culture plates at a density of 5 × 10^3^ cells/well and grown in serum-containing culture medium for 24 h. Varying drug formulations were added, respectively. The concentration of daunorubicin or quinacrine (daunorubicin: quinacrine = 1: 1, μM/μM) was in the 0 - 10 μM range. Culture medium was used as a blank control. After treatment for 48 h, the cytotoxicity was performed by a sulforhodamine B (SRB) staining assay [38]. Finally, dose-effect curves were plotted according to the assay data. Each assay was repeated in triplicate.

### Multifunctional targeting effects

BBB model was established as previously described [39]. Briefly, murine brain microvascular endothelial cells (BMVECs, Institute of Clinical Medical Sciences, China-Japan Friendship Hospital, Beijing, China) were maintained in the medium (DMEM, 20% FBS, 100 units/mL penicillin, 100 units/mL streptomycin, 2 mmol/L l-glutamine, 100 μg/mL endothelial cell growth factor, 40 U/mL insulin). BMVECs were seeded into the gelatin solution coated transwells (Corning, NY, USA; 0.4 μm pore size, 12 mm diameter, and 1.12 cm^2^ surface area) at a density of 3.5 × 10^4^ cells/well, and were incubated at 37 °C for 6 days. Culture medium was changed every other day. Prior to experiment, tightness of the BBB model was evaluated by measuring TEER values. Once the TEER values were higher than 250 Ω·cm^2^, the BBB model was applied for the further experiments.

To evaluate the transport ratio of daunorubicin across the BBB, varying drug formulations were added in the transwells at a concentration of 50 μM daunorubicin. After treatment, a volume of 500 μL sample was taken from basal chamber at 0, 0.5, 1, 1.5 and 2 h. The concentration of daunorubicin was measured using HPLC as above, and transport ratio of daunorubicin was estimated as our previous reports.

To understand the inhibitory effects to GSCs after drug transport across the BBB, the BBB model was formed in the inserts, and then transferred to another 12-well culture plates where GSCs had been cultured for 24 h. Then, varying formulations were added to BBB models, respectively. After incubation for 2 h, the inserts were moved away, and GSCs were further incubated for 48 h. The following procedures were the same with those used in SRB assay.

### Regulating ABC transporters and apoptotic proteins

To evaluate regulating effects of multifunctional targeting drugs-loaded liposomes on the ABC transporters, glioma cells and GSCs were cultured with serum-containing medium for 24 h. Then, glioma cells were treated with blank medium while GSCs were treated with blank medium and with varying drug formulations at a concentration of 10 μM daunorubicin or quinacrine. The cells were further cultured for 12 h. ABC transporter proteins were analyzed using the microplate reader according to manufacturer instructions of the kits (Cusabio Biotech, Co. Ltd., Wuhan, China).

The regulating effects of multifunctional targeting drugs-loaded liposomes on the apoptotic proteins (Fas, Fasl, caspase 8, caspase 9, caspase 3, Bax, Bok, Bcl-2, Mcl-1, and P53) in GSCs were determined using ELISA kits (Cusabio Biotech). Briefly, the cells were cultured for 24 h, and then were treated with varying drug formulations at a concentration of 10.0 μM daunorubicin, respectively. Control experiments were performed by adding culture medium. After incubation for 12 h, cells were harvested and lysed. Cell lysates were analyzed by a microplate reader according to manufacturer instructions of the kits.

### Penetrating ability and destructing effects in glioma stem cell spheroids

After continuous cultured in serum-free medium for 3 weeks, GSCs spheroids were formed. To evaluate the penetrating ability, coumarin was used as the fluorescent probe. Briefly, GSCs spheroids were treated with free coumarin, coumarin liposomes and multifunctional targeting coumarin liposomes for 12 h, respectively. The final concentration of coumarin was 10 μM. After the spheroids were washed with PBS, the samples were observed at the different layers from top to middle of the spheroids using a confocal laser scanning fluorescent microscope [40].

To evaluate the destructing effects of multifunctional targeting drugs-loaded liposomes on the spheroids, GSCs spheroids were collected into six-well culture plates, and then treated with free daunorubicin plus quinacrine, daunorubicin plus quinacrine liposomes and multifunctional targeting daunorubicin plus quinacrine liposomes at a concentration of 10 μM daunorubicin or quinacrine. Blank control was treated with culture medium. After incubation for 48 h, the spheroids were fixed by 2.5% glutaraldehyde for 60 min, washed 3 times with cold PBS, dehydrated and embedded. The spheroids were observed under a scanning electron microscope (SEM, JSM-5600 LV, JEOL, Japan).

### Anticancer efficacy in brain glioma-bearing mice

Imprinting control region (ICR) mice bearing GSCs were prepared as reported previously [35]. All procedures were performed according to guidelines of the Institutional Authority for Laboratory Animal Care of Peking University. Briefly, the mice (18-20 g, Peking University Experimental Animal Center, Beijing, China) were anesthetized (0.2 mL / 20 g) by 4% chloral hydrate and incised through the skin to expose the cranium. The coordinates of the implant target point was set at 1.0 mm anterior from the coronal suture, 3.5 mm right-lateral from the sagittal suture, and 3.0 mm in depth. A volume of 3 μL GSCs (5 × 10^4^ cells/μL) was implanted into target point of each mouse using a stereotaxic instrument (RWD Life Science Co. Ltd., Shenzhen, China). At day 10 post inoculation, the mice were randomly divided into six groups (6 mice/group). Physiological saline, free daunorubicin plus quinacrine, daunorubicin plus quinacrine liposomes, TAM-mediated targeting daunorubicin plus quinacrine liposomes, WGA-mediated targeting daunorubicin plus quinacrine liposomes, and multifunctional targeting daunorubicin plus quinacrine liposomes were administrated via tail vein at a dose of 5 mg/kg, respectively. Treatments were undergone every other day for consecutive 4 times. The survivals of these mice were observed and survival curves were made.

### *in vivo* imaging observation

To evaluate targeting effects in mice, multifunctional targeting DiR liposomes were used as a fluorescent probe [41]. Briefly, 18 male ICR mice were inoculated with GSCs as above. After inoculated for 14 days, the mice were randomly divided into six groups, and administered saline, free DiR, DiR liposomes, TAM-mediated targeting DiR liposomes, WGA-mediated targeting DiR liposomes and multifunctional targeting DiR liposomes (1 μg DiR each) via tail vein. Then, the mice were scanned at 1, 3, 6, 9, 12, 24 and 48 h using a Kodak multimodal imaging system (Carestream Health, Inc., USA). After sacrificed at 48 h, major organs including hearts, livers, spleens, lungs, kidneys and brains (containing tumor masses) of mice were removed immediately. The fluorescence signals in different organs were photographed.

### The specificity to glioma tissues

To evaluate the specificity of the multifunctional targeting liposomes to glioma tissues, coumarin was used as the fluorescent probe. Briefly, 6 mice were inoculated with GSCs as above. After inoculated for 14 days, the tumor-bearing mice were randomly divided into six groups, and administered with saline, free coumarin, coumarin liposomes, TAM-mediated targeting coumarin liposomes, WGA-mediated targeting coumarin liposomes and multifunctional targeting coumarin liposomes (50 μg coumarin each) via tail vein, respectively. After administration for 2 h, mice were sacrificed, and then hearts were perfused with 20 mL PBS for removing the blood in the brain. Brains were removed to make cryosections at 10 mm per slice. The slices were stained with Hochest 33258 (2 μg/mL) for 20 min in the dark and then washed 3 times with cold PBS. The samples were analyzed using a confocal laser scanning fluorescent microscopy (Leica, Heidelberg, Germany).

### Statistical analysis

Data are presented as the means ± SD. One-way analysis of variance was used to determine the significance among groups, after which, post-hoc tests with the Bonferroni correction were used for multiple comparisons between individual groups. A value of P < 0.05 was considered to be significant.

## CONCLUSIONS

A kind of multifunctional targeting daunorubicin plus quinacrine liposomes was developed by modifying with TAM and WGA. The multifunctional targeting daunorubicin plus quinacrine liposomes demonstrated strong capabilities in crossing the BBB, in killing glioma cells and GSCs *in vitro* and in glioma-bearing mice. The action mechanisms for the enhanced efficacy in treating brain glioma are due to the following aspects: (i) evading the rapid elimination from blood circulation by incorporating PEGylated lipid materials on the liposome vesicles; (ii) crossing the BBB via the adsorptive endocytosis mediated by WGA and via blocking the efflux of drugs mediated by TAM; (iii) improving drug uptake by glioma cells and GSCs via the receptor-mediated endocytosis by WGA and via blocking the efflux of internalized drugs from GSCs by TAM; (iv) down-regulating the overexpressed ABC transporters (ABCB1, ABCC1 and ABCG2) that mediated drug resistance of GSCs; (v) inducing apoptosis of GSCs via up-regulating apoptotic receptor/ligand (Fas and Fasl), activating apoptotic enzymes (caspase 8, 9 and 3), activating pro-apoptotic proteins (Bax and Bok), activating tumor suppressor protein (P53) and suppressing anti-apoptotic proteins (Bcl-2 and Mcl-1). Consequently, the multifunctional targeting daunorubicin plus quinacrine liposomes offer a potential strategy for treating brain glioma and GSCs.

## SUPPLEMENTARY MATERIAL FIGURE AND TABLES


