# Supplementary material for: Multifunctional targeting daunorubicin plus quinacrine liposomes, modified by wheat germ agglutinin and tamoxifen, for treating brain glioma and glioma stem cells
Source: Oncotarget. 2014 Jul 26;5(15):6497–511. doi: 10.18632/oncotarget.2267 (PMC4171646; doi:10.18632/oncotarget.2267)
Supplement: Supplementary file 1 [file oncotarget-05-6497-s001.pdf]

Multifunctional targeting daunorubicin plus quinacrine liposomes, modified by wheat germ agglutinin and tamoxifen, for treating brain glioma and glioma stem cells

Supplementary Material

			Size (d.nm):	% Intensity:	St Dev (d.nm):
Z-Average (d.nm):	102.5	Peak 1:	121.1	100.0	53.75
PdI:	0.159	Peak 2:	0.000	0.0	0.000
Intercept:	0.955	Peak 3:	0.000	0.0	0.000
Result quality :	Good				

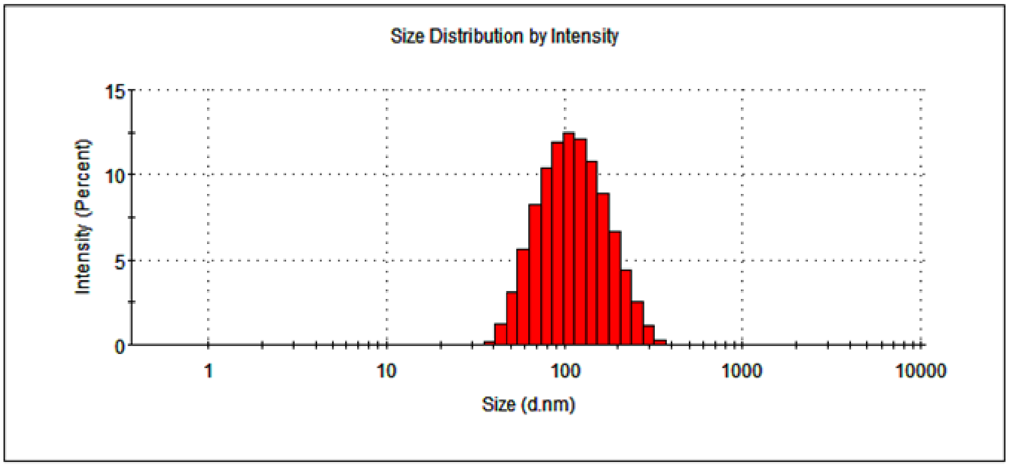

Fig. S1: Particle size distribution of multifunctional targeting daunorubicin plus quinacrine liposomes

Table S1: IC50 values (μM) of varying drugs-loaded liposomes in both glioma cells and glioma stem cells.

Cancer cells	Daunorubicin liposomes	Daunorubicin plus quinacrine liposomes	TAM-mediated targeting daunorubicin plus quinacrine liposomes	WGA-mediated targeting daunorubicin plus quinacrine liposomes	Multifunctional targeting daunorubicin plus quinacrine liposomes
Glioma cells	6.30 ± 0.54	4.53 ± 1.05	3.13 ± 0.36	3.46 ± 0.82	2.23 ± 0.96[a,b,d]
GSCs	9.29 ± 0.57	5.14 ± 1.12	4.77 ± 0.85	3.90 ± 0.40	3.28 ± 0.97[a,b,c]

Notes: $p < 0.05$; a, vs. daunorubicin liposomes; b, vs. daunorubicin plus quinacrine liposomes; c, vs. TAM-mediated targeting daunorubicin plus quinacrine liposomes; d, vs. WGA-mediated targeting daunorubicin plus quinacrine liposomes.

Table S2: Median survival times of tumor-bearing mice after treatments with varying formulations.

groups	physiological saline	Free daunorubicin plus quinacrine	Daunorubicin plus quinacrine liposomes	TAM-mediated targeting daunorubicin plus quinacrine liposomes	WGA-mediated targeting daunorubicin plus quinacrine liposomes	Multifunctional targeting epirubicin plus celecoxib liposomes
Median survival times	26.00	24.00	30.83	34.50[a]	33.67	36.33[a,b]

Notes: $p < 0.05$; a, vs. physiological saline; b, vs. free daunorubicin plus quinacrine.
